# The role of the generalist paramedic in enhanced practice

**DOI:** 10.29045/14784726.2025.6.10.1.71

**Published:** 2025-06-01

**Authors:** Caitlin Wilson, Samantha McCabe-Hogan

**Affiliations:** Yorkshire Ambulance Service; University of Hertfordshire ORCID ID: https://orcid.org/0000-0002-9854-4289; Sheffield Hallam University

We are writing to highlight the evolving role of the generalist paramedic, particularly in the context of enhanced practice and portfolio careers. While much attention has been paid to specialist paramedic career pathways in primary care, urgent/community care, critical care and mental health, the value and complexity of generalist practice remain underexplored. Generalist paramedics maintain a broad clinical scope, while incorporating elements of research, education and leadership into their practice.

The recently published *Enhanced Level Practice* schemas for Allied Health Professionals (NHS England, 2024) provides a structured pathway for paramedics to develop their roles while remaining in generalist practice. Enhanced practice represents a highly valued level within the healthcare workforce, characterised by experienced registered professionals who deliver complex care, manage day-to-day risks and make substantial contributions across diverse settings. Often positioned as a precursor to advanced practice, enhanced practice is increasingly recognised as both a stepping stone and a valued career destination in its own right.

The *Paramedic Career Framework* (College of Paramedics, 2024) emphasises the flexibility for paramedics to develop across the four pillars of clinical practice, leadership, education and research. Notably, the framework identifies the role of the specialist/enhanced paramedic as a senior position within the clinical practice pillar while offering development opportunities in other areas, such as education or research. This balance allows paramedics to shape their career according to individual strengths and interests, supporting both generalist and specialist pathways. This perspective is particularly relevant to paramedics embracing portfolio careers, as generalism itself may appeal to those prioritising education or research as their main focus.

Despite these advances, recognition and career pathways for generalist paramedics remain inconsistent. Generalist practice requires enhanced decision making, adaptability and holistic patient care. However, the narrative often underestimates the expertise required to manage the diverse needs of patients presenting to the ambulance service, risking the marginalisation of generalist contributions. A recent College of Paramedics *INSIGHT* article by McCabe-Hogan (2024) underscores this point, urging that enhanced practice roles must avoid being perceived as exclusive to clinical specialists. Instead, generalism should be celebrated as a distinct and valuable area of enhanced practice in its own right.

We hope this letter will spark further discussion on the role of the generalist paramedics in enhanced practice.

Yours sincerely,

Dr Caitlin Wilson and Samantha McCabe-Hogan

## Acknowledgements

We are grateful to Dr Fiona Bell for her input into early ‘generalist paramedic’ discussions that led to the writing of this letter.

## Author contributions

CW and SMcH conceived the letter. CW drafted the first version of the letter. Both authors contributed substantially to its revisions and approved the final submission. CW acts as the guarantor for this article.

## Conflict of interest

CW is an associate editor of the *British Paramedic Journal*.

## Funding

None.
